# Theory-driven development of an educative nutritional intervention (ENI) supporting older hospital patients to eat sufficiently, assisted by an eHealth solution: an intervention mapping approach

**DOI:** 10.1186/s12913-022-08679-8

**Published:** 2022-11-28

**Authors:** Rikke Terp, Tove Lindhardt, Lars Kayser

**Affiliations:** 1 Department of Internal Medicine, Copenhagen University Hospital, Herlev and Gentofte Hospital, Hospitalsvej 1, 2900 Hellerup, Denmark; 2 Department of Public Health, Section for Health Service Research, University of Copenhagen, Øster Farimagsgade 5, 1353 Copenhagen K, Denmark

**Keywords:** Malnutrition, Older patients, Patient participation, Self-management, eHealth, eHealth literacy, Educative nutritional intervention, Intervention mapping

## Abstract

**Background:**

Insufficient protein and energy intake is a prevalent and serious problem in older hospital patients. Here, we describe the development of a program consisting of **1**) an educative nutritional intervention (ENI) to support older hospital patients to participate in their own nutritional care using the eHealth solution Food’n’Go, and **2**) a plan for education and support of healthcare professionals, enabling them to conduct the ENI. Further, we describe the evaluation of the acceptability of the program as perceived by nursing staff and dieticians.

**Methods:**

The Intervention Mapping (IM) framework was used to design and develop the ENI through six steps: **1**) a logic model of the problem was developed; **2**) performance objectives and related change objectives were defined for patients, relatives, and healthcare professionals; **3**) the intervention was designed using relevant theory-based change methods; **4**) program materials were produced; and finally, **5)** implementation and maintenance were planned and **6**) evaluation of the program was planned. End users (patients, relatives, and healthcare professionals) were involved in the design and development of the ENI.

**Results:**

Based on the logic model, the personal determinants (knowledge, skills, self-efficacy, outcome expectation, social support, attitude, and awareness) related to the patients and their relatives were addressed in the ENI, and those related to the healthcare professionals were addressed in the plan for their education and support. Theories of behavioral change, technology acceptance, and nutritional management for older persons were applied. A plan for evaluation of the effectiveness (intake of energy and protein) and feasibility of the ENI was conducted. The feasibility measurements were the behaviors and determinants related to the intervention outcome that were identified in the logic model of change. The ENI was perceived as acceptable by the nursing staff and dieticians.

**Conclusion:**

We developed a theory- and evidence-based intervention guided by the IM framework and a sociotechnical approach, which was perceived as acceptable and ready for use to support older hospital patients to eat sufficiently assisted by eHealth.

**Supplementary Information:**

The online version contains supplementary material available at 10.1186/s12913-022-08679-8.

## Background

The risk of protein-energy malnutrition is prevalent in older hospital patients, with estimates reaching 53% in Europe [1]. Hospitalization increases the risk of malnutrition due to decreased food intake combined with increased energy needs during acute illness [2]. Insufficient nutritional intake in adult patients imposes additional risks, as malnutrition is associated with functional decline [3], prolonged hospitalization [3, 4], increased risk of re-admission [3–5], and increased mortality [4, 6–8]. Several interventions aimed at preventing malnutrition in older patients have shown improved energy and protein intake, while the results on functional outcomes are inconsistent [2, 9–12]. Older patients are a heterogeneous group in terms of reasons for insufficient dietary intake, nutritional needs, and food preferences. Therefore, in accordance with the recommendations from the European Society for Clinical Nutrition and Metabolism (ESPEN), nutritional interventions for older people should be individualized and comprehensive [13]. With the increasing digitalization of healthcare services, technology may facilitate a more individualized approach. However, evidence on the use of eHealth for older patients and nutritional care is sparse [14].

Previously, our research group developed a tablet-based eHealth solution, Food’n’Go, designed to engage older patients and their caregivers in a joint effort to ensure that patients eat adequately [15]. Food’n’Go was made available in two hospital units in 2017. During hospitalization, patients are provided with a computer tablet with an application allowing them access to the food and drink menu in the hospital. They can then order and register their food themselves and receive feedback as to whether their food intake meets their dietary requirements for energy and protein. However, observations in 2018 showed that only 25% (6 out of 24) of the patients were informed about and used Food’n’Go, despite it being observed that 54% (13 out of 24) were able to use it (unpublished data, can be provided by Terp R). We hypothesized that Food’n’Go combined with an educative nutritional intervention (ENI) addressing older patients’ needs, competencies, and preferences for being actively involved in their own nutritional care and for using eHealth would motivate and support them to participate in efforts to eat adequately.

In this study, we used the Intervention Mapping (IM) framework by Bartholomew et al. [16] to develop the ENI. IM guides the design and development of evidence- and theory-based behavioral change interventions. IM has been widely used to develop health promotion interventions, including in hospital settings [17, 18]. Positive findings have been reported by other authors who have used IM to develop behavioral nutrition interventions [19, 20] and in studies developing eHealth interventions [21, 22]. IM is based on a social ecological approach that guides the program planner to understand the intervention as part of a system in which individuals and environmental factors are interrelated, and must therefore be addressed when developing interventions. This understanding of health interventions is important when developing an intervention such as the ENI because (1) insufficient dietary intake in older patients may be caused by multiple individual end environmental factors, and (2) a sufficient intake in this patient group often depends on support from nursing staff and relatives.

The ENI is intended to involve nurses, dieticians, and physicians; however, the main focus of involvement is the nursing staff. Nurses play a key role in the nutritional care of older patients, as they are responsible for performing nutritional screening, observing food intake, ensuring access to food and eating support, and involving patients, thereby empowering and motivating them to eat sufficiently [23, 24]. Here we present the methods used for designing and developing an intervention program consisting of the patient-directed ENI and nursing staff-directed education and support, guided by IM. Further, we report the results of the acceptability of this program as perceived by healthcare professionals.

## Development of the intervention map

### Setting

In Denmark, all citizens have free access to hospitals. They are referred by their general practitioners, other medical specialists, acute clinics, or emergency services. This study took place in a hospital unit specializing in infectious diseases, with 21 beds and an average length of stay of 5.5 days. The average age of the patients in 2019 was 75 years. The current standard procedure for nutritional care instructs nursing staff to screen patients for malnutrition using nutritional risk screening (NRS-2002) within 24 h of admission [25]. The nursing staff includes registered nurses (RNs) and licensed practical nurses (LPNs). The standard nutritional care offered to patients who are at risk of malnutrition includes monitoring their daily nutritional intake, and if they are not able to reach their required energy and protein needs, they may be referred to a dietitian for individual dietary counseling. All nutritional services are free of charge for patients.

In the hospital unit studied, the eHealth solution Food’n’Go was made available to all patients in 2017. The nursing staff is expected to support patients in using Food’n’Go. A project nurse was employed in the unit to facilitate the implementation of the eHealth solution from 2018 to the end of 2019. The ENI described in this article was developed to support patients’ participation in their own nutritional care using Food’n’Go. The resulting intervention reported here was introduced at the end of February 2020. Due to the COVID-19 pandemic, the intervention was paused in March and April 2020 and was reintroduced later and evaluated after three months. The first and second authors are RNs with several years of experience in geriatrics and nutrition, and are employed in the hospital department where the participating unit is situated. The first author holds a PhD, and the second author is a senior researcher and head of the Food’n’Go research program. The last author is a medical doctor and associate professor at the University of Copenhagen, Section for Health Service Research, and has worked with research and education in the field of health care innovation for more than 10 years.

### The intervention mapping framework

IM describes the systematic process and associated tasks for design, implementation, and evaluation in six steps: (1) develop a logic model of the problem, (2) develop matrices of change objectives, (3) design the intervention, (4) produce the components of the intervention program, (5) plan implementation of the intervention program, and (6) plan the evaluation of the intervention. Each task informs the subsequent step in an iterative process [16, 26]. We present the six steps in three parts: Part A describes the method and results of steps 1 to 4; Part B describes the implementation, maintenance, and evaluation process (steps 5 and 6); and Part C describes the results of step 6 regarding the acceptability of the intervention program as perceived by the nursing staff, the nurse manager, and the dietician.

## Part A: Identification of problems and needs, and creation of plan and materials in steps 1 to 4

## Step 1: logic model of the problem, the needs assessment

### Method

In this step, we conducted a needs assessment to develop a logic model of the problem describing (1) the health problem, which was insufficient food intake in older (≥ 65 years) hospitalized patients; (2) the behavior of the patients, relatives, and healthcare professionals, and the environmental factors leading to insufficient food intake; and (3) the personal determinants of the behavioral factors [16, 26].

#### Data sources

Our needs assessment builds on the existing literature regarding behavioral and environmental factors influencing insufficient food intake in older people [2, 27–32] and personal determinants of the adoption and use of technology [33, 34]. To obtain evidence of older patients’ competence, preferences, and attitudes toward nutrition and technology, we initially conducted an explorative study. We also conducted an observational study to assess older patients’ competence and use of Food’n’Go in this hospital unit and used empirical data to describe local practice, context, and workflows.

#### Older patients’ competence, preferences, and attitudes toward nutrition and technology

The explorative study was conducted in 2018 using semi-structured interviews and a questionnaire (the Readiness and Enablement Index for Health Technology) with 25 older patients (aged ≥ 65 years). The patients were recruited from two hospital units specializing in infectious diseases, one of which was the participating unit when developing the ENI. Patients with terminal illness, inability to give informed consent due to cognitive or physical impairment, or poor comprehension of the Danish language were excluded. The qualitative data set was separated and analyzed into a “technology part” and a “nutrition part.” The overall design has been reported elsewhere [31, 35].

#### Patients’ use of Food’n’Go

The observational study was conducted in 2018 to assess (1) patients’ need for support in the use of Food’n’Go and (2) current nursing practices in the hospital unit for involving and supporting patients in the use of Food’n’Go. On two different weekdays within a week, all patients in the unit were screened for eligibility to participate. We excluded those who were unable to give informed consent or did not eat orally. The included patients were interviewed about their use of Food’n’Go, and afterwards, they were asked to complete specific tasks on the tablet using Food’n’Go, such as ordering lunch and registering their food intake from the previous meal. An interview and observation guide were developed, and a pilot involving four patients in two iterations led to an additional item asking for reasons for the non-use of Food’n’Go. If additional information regarding the patients’ needs for support emerged, it was documented in the field notes.

#### Local practice, context, and workflow

To identify healthcare professionals’ concerns and needs in relation to the planned intervention, we gathered data about their behaviors and attitudes regarding nutrition and Food’n’Go. Data were collected from project group and staff meetings, and an interview with the project nurse who participated in the implementation of Food’n’Go. We also received statistics on the usage of Food’n’Go from the IT company Movesca. A project group had already been established in the Food’n’Go project, whose goal was to ensure effective implementation of the technology by discussing and planning necessary adjustments to the Food’n’Go technology.

### Results

**Fig. 1:**
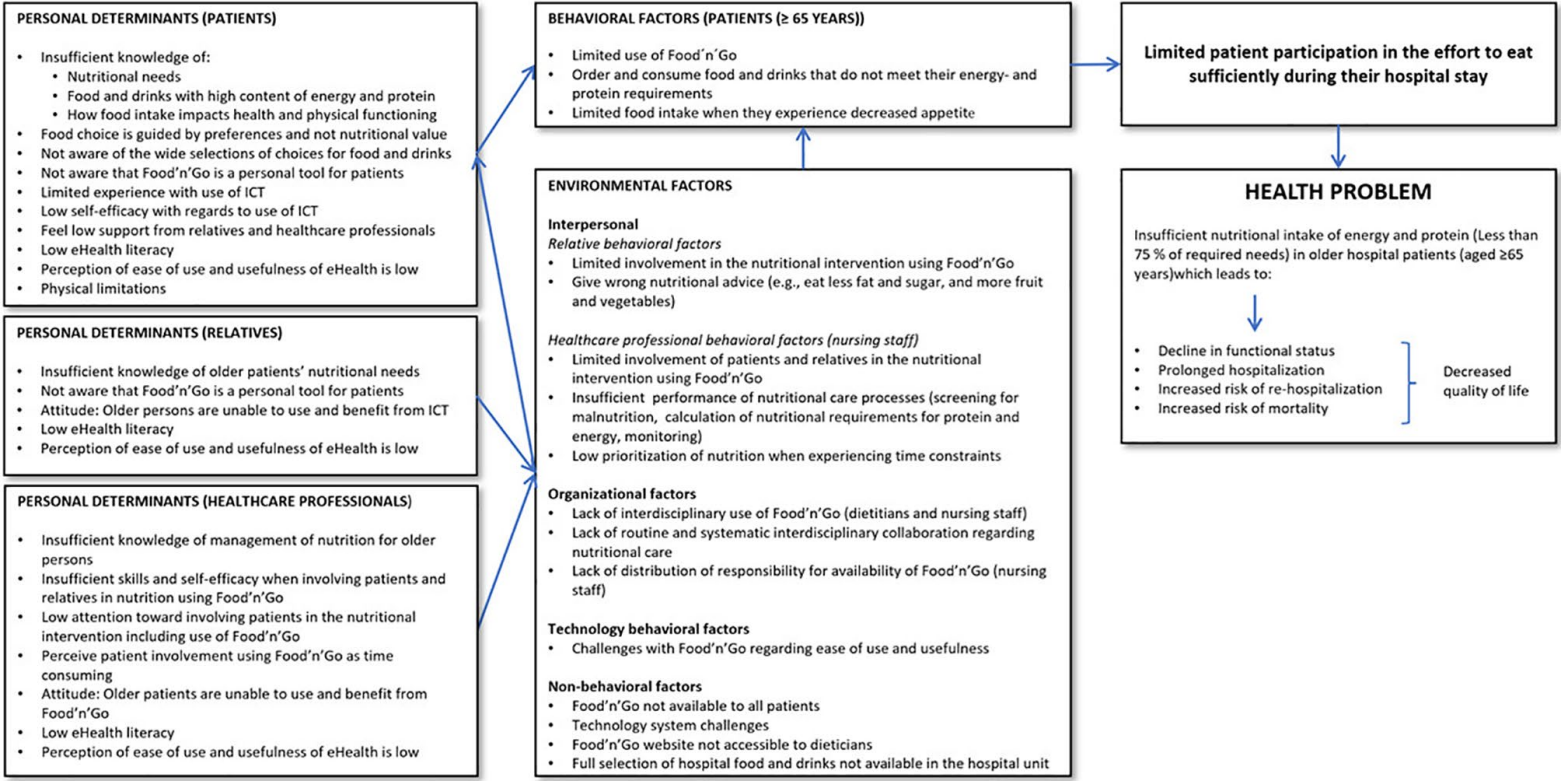
The needs assessment—Logic model of the problem (step 1)

The needs assessment is shown in a logic model of the problem in Fig. 1. The needs assessment established that the intervention should target patients and their relatives as well as healthcare professionals.

#### Theory, evidence, and empirical findings

Based on theory, evidence, and empirical findings [2, 30–32, 34–37], we identified behavior and related personal determinants among patients, relatives, and healthcare professionals, as well as non-behavioral factors that may lead to insufficient nutritional intake and lack of use of Food’n’Go. We supplemented the empirical findings with theory to refine the identified personal determinants of using (1) the Readiness and Enablement Index for Health Technology (READHY) framework [34], which addresses determinants of readiness to engage with eHealth, and (2) the Technology Acceptance Model 3 (TAM3), which describes determinants of individuals’ acceptance and use of information technologies [33]. The empirical findings that were used in the logic models are summarized below.

#### Older patients’ competence, preferences, and attitudes toward nutrition and technology

The findings from the explorative study are reported in two articles: one reporting how food and nutrition are understood by older patients [31] and the other reporting on digital competence and readiness [35]. In this section, we summarize the main findings that were included in the logic model. The participants (older patients) were, in general, motivated to engage in activities that improved their health. However, they seemed to lack knowledge about their nutritional needs when older and acutely ill and the association between their food intake and their health. They also had limited experience with monitoring their nutritional status [31]. We found that their experience with technology ranged from not using information and communication technology (ICT) at all to advanced usage, which was reflected in a wide range of competences, preferences, and attitudes toward ICT. Non-use was often related to a lack of knowledge and low self-confidence about the use of ICT [35].

#### Patients’ use of Food’n’Go

In the observational study, we screened 29 patients for eligibility to participate, and 9 (mean age 85 years, SD: 5.2) were excluded due to the following reasons: unwilling to participate (n = 1), unable to provide informed consent (n = 2), no oral food intake (n = 3), unavailable due to examinations or discharge before inclusion (n = 3). In total, 20 patients were included, with a mean age of 76 years (SD: 14.2). The five pre-defined categories for patients’ level of need for support were condensed into four categories, with the included patients distributed as follows: (1) Patient is able to complete the tasks on the tablet with no need for support (n = 7; mean age: 62 years (SD: 9.7)); (2) Patient is able to complete the tasks on the tablet with support (n = 3; mean age: 73 years (SD: 8.0)); (3) Patient is able to participate in completing the tasks when the tablet is held and operated by another (n = 10; mean age: 86 years (SD: 8.7)); or (4) Patient is not able to participate in completing the tasks (n = 0).

We presented the above findings to the nursing staff and encouraged them to discuss whether additional categories should be added. The nursing staff agreed with the above categories. This study demonstrated that only a limited number of patients participated in nutritional care using Food’n’Go. Three out of ten patients in categories 1 or 2 (i.e., able to hold and operate the tablet with Food’n’Go) used it. The following findings were included in the logic model: nursing staff did not systematically introduce patients to Food’n’Go, and patients were unaware of the possibility of using Food’n’Go as a personal tool, as they thought it was only to be used by the nursing staff.

#### Local practice, context, and workflow

Local audits and statistics provided by the IT company Movesca confirmed an insufficient intake of energy and protein (less than 75% of estimated requirements) among patients. Data from project and staff meetings and interviews with the project nurse confirmed the limited involvement of patients in using Food’n’Go and revealed related barriers. A predominant attitude among the nursing staff was that older patients were not capable of using and benefiting from eHealth. This, in combination with technical challenges in the early version of Food’n’Go, was identified as a significant determinant for not involving patients in its use. The nursing staff perceived patient involvement as time-consuming, and some lacked trust in the technology due to previously mentioned challenges (e.g., system failure and problems with internet access). Furthermore, limited experience, skills, and knowledge in supporting patients to participate in using Food’n’Go contributed to the nursing staff’s failure to involve patients. Local audits showed that the nursing staff’s completion of the required procedures for nutrition management, such as screening for malnutrition and calculating nutritional requirements, was suboptimal, and this was a barrier to the successful use of Food’n’Go. A lack of systematic interdisciplinary collaboration in the nutritional care in older patients was included in the model.

## Step 2: logic model of change

### Method

In this step, we defined the target behavior of patients, relatives, and healthcare professionals and the related personal determinants and non-behavioral environmental factors required to reach the intervention goals. The intervention goals were defined as (1) daily energy and protein intake reaching at least 75% of patients estimated requirements, and (2) patients’ participation in nutritional care using Food’n’Go. We created matrices linking the target behavior (i.e., the performance objectives) with the determinants and related change objectives, specifying what needed to be changed. Based on social cognitive theory (SCT), we categorized the personal determinants into six categories: knowledge, skills, self-efficacy, outcome expectation, social support, and attitude [38, 39].

### Results

The matrices visualized what needed to be addressed in the educative nutritional intervention (ENI) and in the program for education and support targeting healthcare professionals to achieve the intervention goals. The performance objective for the patients was tailored to the four defined patient profiles describing their competence and need for support in using Food’n’Go. An example of a performance objective linked to change objectives targeting the patients is shown in partial matrices in Table 1 (*complete matrices are attached as Additional file 1*). Performance objectives required for the patients to achieve the intervention goals were as follows: (1) use Food’n’Go (i.e., participate in ordering and monitoring their own food intake, and adjustment of their food intake according to the feedback provided from Food’n’Go); and (2) order and eat food that meets their nutritional needs. Performance objectives for relatives addressed how they could support and motivate the patients by talking about their food intake, encouraging them to order and eat between meals, keeping them company during meals, and assisting them in the use of Food’n’Go. Examples of performance objectives for the nursing staff were (1) inform and introduce Food’n’Go to patients according to their needs and competence, and (2) provide personal feedback to the patients about their nutritional intake using the feedback diagram in Food’n’Go. Non-behavioral environmental performance outcomes were (1) Food’n’Go is available to all patients, (2) patients and healthcare professionals experience only a few technology challenges, (3) the dieticians are granted access to Food’n’Go, and (4) food and drinks visible to the patients in Food’n’Go are available in the hospital unit. An overview of change objectives for all target groups is provided in complete matrices, attached as Additional file 1.

**Table 1:** Partial matrices of change objectives for patients (step 2)

Performance objectives (PO) for patients	Change objectives						
	**Knowledge**	**Skills**	**Self-efficacy**	**Outcome expectation**	**Social support**	**Attitude and awareness**	
Profile 1 and 2* PO.1 **Use of Food’n’Go** Operate the tablet and use Food’n’Go • Order food and drinks • Register food intake** • Monitor and adjust food intake if necessary	Express the routines with regard to using Food’n’Go to order food and register their intake – when and how	Demonstrate skills to operate Food’n’Go to: • Order food • Register intake • Adjust food intake based on feedback from Food’n’Go to ensure a sufficient dietary intake	Express confidence in ordering food and registering their food intake in Food’n’Go	Expect Food’n’Go to provide options to choose tasty foods that will meet their preferences and dietary requirements Expect the healthcare professionals and relatives approve of they use Food’n’Go	Perceive the nursing staff provides them with necessary support with regards to using Food’n’Go Ask the nursing staff for help when needed with regards to Food’n’Go Perceive support from their relatives in using Food’n’Go	Perceive Food’n’Go to be useful and easy to use	
Profiles 1, 2, and 3* PO.2 Order and eat food that meets their nutritional needs	Express their nutritional needs Express how a sufficient food intake positively affects their health, including physical functioning Express they need food and drink with high content of energy and protein Identify food and drink items with high content of protein and energy	Demonstrate use of feedback on their daily food intake provided in Food’n’Go and adjust their food intake during the day, if necessary		Expect that eating sufficiently will positively affect their recovery and physical function Expect that they will be served well-prepared and tasty food Expect the healthcare professionals and relatives will approve that they eat sufficiently during their hospital stay	Perceive support from their relatives and that they are engaged in the nutritional care Perceive that the healthcare professionals will provide them with necessary support with regards to selecting food that meets their needs	Aware of the importance of eating despite no or decreased appetite	

* Profile 1: Patient is able to hold and operate the tablet with Food’n’Go without support

Profile 2: Patient is able to hold and operate the tablet with Food’n’Go with verbal and/or technical support

Profile 3: Patient is able to participate in completing the tasks when the tablet with Food’n’Go is held and operated by another

** Only required in patients at risk of malnutrition (NRS > 3)

## Step 3: Designing the intervention: evidence- and theory-based methods and practical applications

### Method

The product of step 3 was a plan of the ENI and a plan of the change objectives for each target group organized by the determinants (skills, self-efficacy, outcome expectations, etc.), linked to theory-based change methods and a description of how the intervention would be delivered in practice. In the design of the intervention, we applied theories of behavioral change [38–42], including SCT by Bandura, describing the influence of self-efficacy and outcome expectations on health behavior [38, 39]. We used the theory of persuasive communication, that is, the elaboration likelihood model of persuasion, to choose methods for changing attitudes [42]. To address the determinants related to the adoption and use of technology, we used the TAM3 by Venkatesh et al., which describes suggestions for interventions targeting the determinants of adoption and use of technology: perceived ease of use and perceived usefulness [33]. The recommendations for nutritional care for older patients addressed in the recent ESPEN guideline (2019)[13] were considered and included in the ENI. To ensure that the end user perspective was accounted for and to facilitate the implementation and adoption of the intervention, representatives of all target groups (patients, relatives, and healthcare professionals) were involved in the creation of and reflection on ideas for the ENI [43]. They were presented with the identified target behavior defined in the logic model of change (e.g., healthcare professionals inform patients and relatives about the benefits of eating sufficiently). Afterwards, they were encouraged to elaborate on these and come up with ideas for practical delivery (e.g., how to provide patients and relatives with nutritional information). The ideas were generated from individual conversations with patients (n = 3; mean age: 80 years), relatives (n = 2; spouses), nurses (n = 2), the nurse manager, the clinical nurse specialist, and the project nurse, as well as one workshop with patients and nurses (n = 4) and group conversations with nursing staff, dietitians, and physicians in four meetings.

### Results

**Fig. 2:**
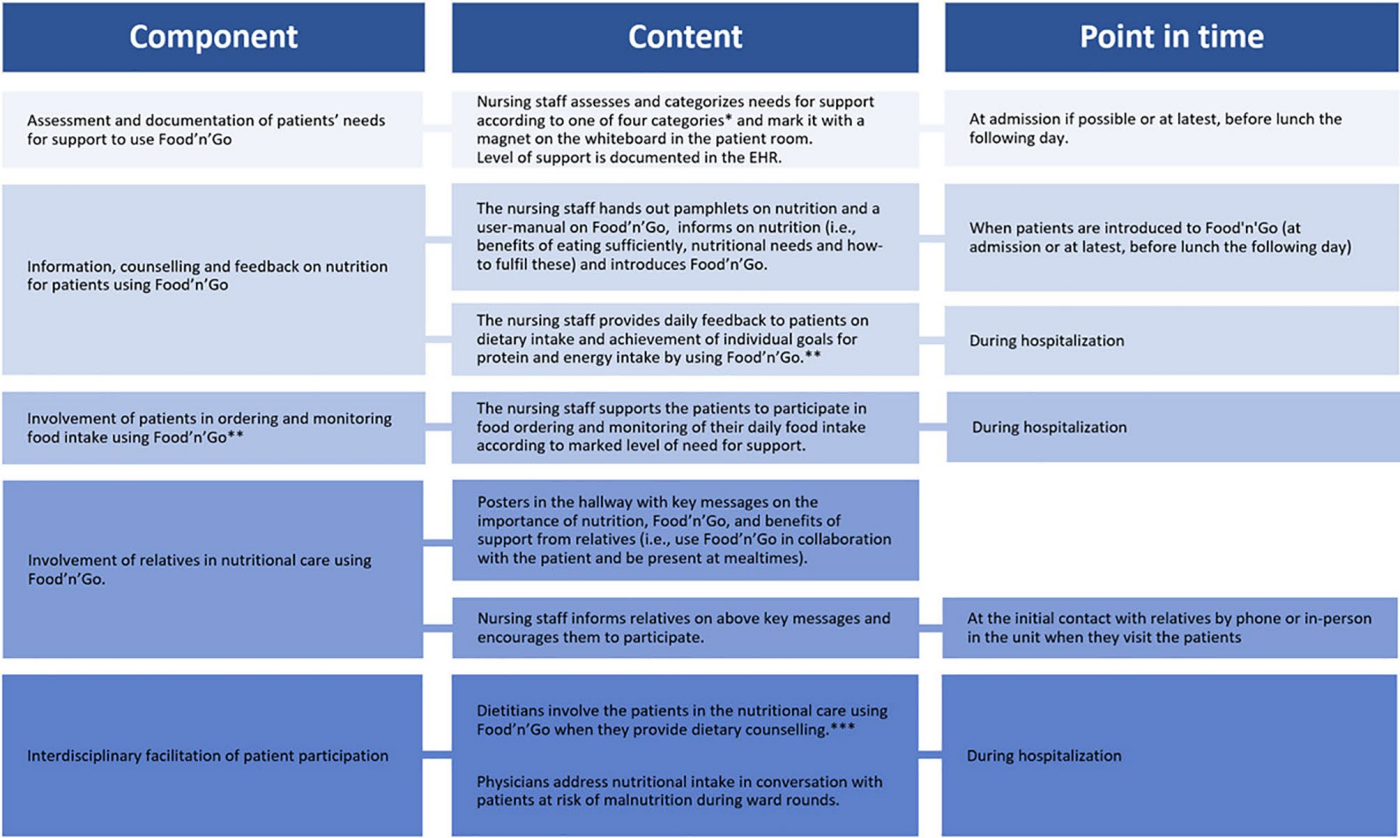
The Educative Nutritional Intervention—ENI. * (1) Patient is able to hold and operate the tablet with Food?n?Go without support; (2) Patient is able to hold and operate the tablet with Food’n’Go with verbal and/or technical support; (3) Patient is able to participate in use of Food’n’Go when the tablet is held and operated by another; (4) Patient is not able to participate in use of Food’n’Go. ** Monitoring of food intake was only required for patients at risk of malnutrition (NRS≥3). *** Dietary counselling by dietitian for patients at risk of malnutrition was a part of standard nutritional care, and was in this ENI extended to include use of Food’n’Go

In collaboration with patients, relatives, and healthcare professionals, we conceptualized and designed the ENI targeting patients and their relatives, and a program for education and support for the healthcare professionals. The ENI consists of five components, which are shown in Fig. 2.

The theory-based change methods and practical applications used to achieve the change objectives for all target groups are shown in Table 2 (*detailed version of* Table 2*is provided as Additional file 2*). Input from patients and relatives guided us to avoid too much written information, avoid lecturing, and minimize the focus on negative consequences, instead emphasizing on the benefits of eating adequately as key messages of the information. The nursing staff requested a systematic and simple approach to categorizing patients at admission with regard to their need for support in using Food’n’Go. They suggested a system with magnets on each patient’s whiteboard that represented their need for support. The dietitians and the physicians contributed to the ENI with requests and suggestions for the communication workflow, such as, how the physicians should receive information on which patients to inform about nutrition.

**Table 2:** Intervention program with theory-based change methods and practical applications (step 3)

Change objectives -	Theory-based change methods	Practical application and delivery
**Patients**		
Knowledge	Provide information ^a,^	• Posters are positioned at the entrances and at the nurses’ station. • Pamphlets are handed out to patients and relatives. • Individual communication with the nursing staff and dietitians takes place. • Food’n’Go user guides are placed on the patients’ whiteboards. • Food’n’Go provides nutritional information (energy and protein content in food and drinks).
Skills	Guided practice ^b, c^ Goal and self-monitoring ^a, b, c^	• The nursing staff instruct patients in how to use Food’n’Go on the day of admission or next day before lunch. Procedures and teaching principles are outlined in guidelines. • Patients at risk of malnutrition (NRS score ≥ 3) are trained to monitor dietary intake and use the feedback provided by Food’n’Go.
Self-efficacy	Guided practice ^b, c^ Encourage and provide feedback on their performance ^c^	• Patients are instructed and supported in the use of Food’n’Go in accordance with their competencies and need for support (according to the four defined patient profiles) and guided toward a mastery experience. • The nursing staff and dietitians use Food’n’Go in guidance and counseling.
Outcome expectation	Provide information on consequences and outcome ^a^ Persuasive communication ^a, d^ Social processes of encouragement ^a^	• Verbal and written information communicates key messages about beneficial outcomes of eating adequately and using Food’n’Go. • During rounds, the physicians inform and talk about nutrition with patients at risk of malnutrition. • The healthcare professionals approve of the patient’s efforts to eat adequately using Food’n’Go.
Social support	Social processes of encouragement and support ^a,^	• Support from relatives is mobilized by providing the relatives with knowledge (see below under ”relatives”).
Attitude and awareness	Persuasive communication ^a, d^ Cues ^a^ Self-monitoring ^a^	• Providing patients with the knowledge and skills described above is expected to influence their attitude and awareness. • Patients at risk of malnutrition are offered individual dietary counseling by a dietitian. • Magnets on the patients’ whiteboard visualize the expected behavior regarding the use of Food’n’Go. • Food’n’Go enables the patients to monitor their food intake themselves.
**Relatives**		
Knowledge	Provide information ^a^	• Written information (posters and pamphlets with key messages about nutrition; a user guide for Food’n’Go) and verbal information are provided by the nursing staff.
Outcome expectation	Provide information on consequences and outcome ^a^	• Information is provided as described above under “knowledge.”
Attitude	Persuasive communication ^a, d^	• Providing the relatives with the above information is expected to influence their attitude. • During personal communication, the nursing staff emphasize the importance of an adequate intake in older people and the benefits of using Food’n’Go.
**Nursing staff Activities provided during intervention period to facilitate implementation**		
Knowledge	Provide information ^a^	• Teaching sessions once a week with different topics related to the ENI. • Individual teaching sessions. • Weekly electronic newsletter. • A guideline describing procedures for conducting the ENI. • Posters with key messages related to the ENI formulated as the “10 nutrition commandments”.
Skills and self-efficacy	Guided practice ^b, c^ Modeling ^a, c, e^ Encourage and provide feedback on their performance ^c^	• Individual bedside teaching with skills training takes place, followed by feedback. • The nutritional key person demonstrates use of the ENI in daily nursing care and acts as role model. • Results from the continuous monitoring are communicated to the staff.
Outcome expectation	Provide information regarding outcome ^a^ Encourage and provide feedback on their performance ^c^	• Weekly teaching sessions are held. • The nursing staff are provided with results from monitoring of the patients’ use of Food’n’Go and their food intake. • The nurse manager: • Participates in meetings and teaching sessions to signify the importance. • Requests need for action in relation to delivery of the ENI.
Attitude and awareness	Persuasive communication ^a, d^ Encourage and provide feedback on their performance ^c^ Facilitation—reduce barriers to action ^f^	• High involvement is facilitated in teaching subjects by providing the nursing staff with the above information about nutrition and patient involvement and encouraging discussion and reflection about the use of ENI during the weekly teaching sessions. • Continuous skills training of skills related to the ENI is expected to change their attitude. • Food’n’Go is available (charged and logged in) to all patients. Weekly checks take place of availability of Food’n’Go.
**Dietitians Activities provided during intervention period to facilitate implementation**		
Knowledge, skills, and self-efficacy	Provide information and instruction ^a^ Persuasive communication ^a, e^ Provide information regarding outcome ^a^ Encourage and provide feedback on their performance ^c^	• Regular meetings take place with the dietitians and first author. • The dietitians participate in the weekly teaching sessions with the nursing staff.
**Physicians Activities provided during intervention period to facilitate implementation**		
Knowledge, outcome expectation, and awareness	Provide information ^a^	• Information meetings are held before the start of the intervention. • At the morning meetings, the interdisciplinary staff are informed and encouraged to perform the required tasks related to the ENI.

^a^Mitchie et al., 2008 [40]; ^b^Kok et al., 2016 [41]; ^c^Kelder et al., 2016 [39]; ^d^Perloff, 2017 [42]; ^e^ Bandura, 2012 [38]; ^f^ Bartholomew et al., 2016 [16]

## Step 4: production of program components

### Method

In an iterative process, we produced, tested, and adjusted the program materials. We presented patients, relatives, and healthcare professionals with the program materials and received their feedback and reflections for adjustments and final approval. We collected the feedback during individual conversations with patients admitted to the hospital unit (n = 3; mean age 82 years), nurses (n = 5), dieticians (n = 2), the nurse manager, and the clinical nurse specialist, and from group discussions in two staff meetings with the nursing staff (n = 14), one meeting with the physicians (n = 5), and two meetings in the Food’n’Go project group. An event was also held in which the first author positioned herself in the hallway, presented the pamphlet and two different versions of posters, and had spontaneous conversations with passing patients, relatives, and healthcare professionals.

### Results

#### Development of program materials

We developed the following materials: **(1) information for patients and relatives**: poster and pamphlet with key messages of the ENI and a user guide for Food’n’Go; **(2) categorization of patients’ needs for support**; a system with four magnets with different labels and colors to categorize the patients’ need for support to use on the whiteboards in the patients’ rooms; **(3) nursing documentation**: a template in the electronic health record (EHR) for documentation of the patients’ need for support; **(4) guidelines for nursing practice**: a description of the required nursing practices related to the ENI including workflows; and **(5) educational material designed for the nursing staff**. We adjusted the materials in several iterations until approval was obtained from the target groups and relevant stakeholders ( the nurse manager, assistant nurse manager, clinical nurse specialist, and project nurse). Adjustments based on feedback from patients and relatives included a more eye-catching poster design and linguistic changes in the pamphlet. The template for nursing documentation developed in the EHR was adjusted in several iterations to create accurate, intuitive, and easy-to-use documentation and avoid a heavy workflow, which is a known barrier for nursing documentation [44].

#### Program for education and training of nursing staff

The nursing staff were mainly responsible for carrying out the ENI; therefore, they were the primary target group for education and training. During the three-month intervention period, the nursing staff were scheduled to receive education and training that targeted the defined change objectives (i.e., self-efficacy, knowledge, and skills to involve patients in the nutritional intervention). Once a week, a joint teaching session for nursing staff and dietitians was held on different topics related to the ENI using different teaching methods, including group discussions about solutions to optimize the delivery of the ENI, feedback on the progress of the intervention, and information provided in an entertaining way, such as quizzes.

In addition to these group meetings, individual teaching sessions were held with nursing staff to practice their skills and increase their self-efficacy of involving patients in the nutritional care using Food’n’Go. In these sessions, the nurse or LPN would (1) receive detailed information on the components in the ENI, (2) practice selected tasks from the ENI under guidance, and (3) receive feedback on their performance of the tasks. These sessions were conducted by the first author, who was present in the unit several days a week to observe the use of the ENI and offer spontaneous support and guidance if needed during the intervention period. Several supportive activities were planned to be conducted by the management team (nurse manager and assistant nurse manager), the clinical nurse specialist, and the nutritional key person, who was an experienced nurse from the hospital unit with special responsibilities for nutrition. The nurse manager addressed the need to focus on the ENI at the morning interdisciplinary meetings, while the key person acted as a role model for staff in use of the ENI. Every week, essential key messages concerning the ENI were communicated to the healthcare professionals in the weekly electronic newsletter.

## Part B: planning implementation, maintenance (step 5), and evaluation (step 6) of the intervention program

For step 5, we report the plan of work processes ensuring implementation and maintenance of the ENI, and for step 6, we report adjustments in the education of healthcare professionals and the plan for evaluation of the ENI. The results of the evaluation regarding the clinical outcomes will be reported elsewhere.

## Step 5: planning the implementation and maintenance of the intervention program

The Food’n’Go project has already been adopted at the leadership level prior to the implementation of the ENI, as Food’n’Go had been iteratively developed in a joint research-based effort between the hospital and the commercial partner Movesca. The ENI was developed as an educative intervention to support the adoption and sustainability of the everyday use of Food’n’Go, which was already endorsed by the head nurse and the nurse manager. To ensure ownership and active and continuous involvement of the management team, the ENI was created together with the nurse manager, clinical nurse specialist, key person, and first author (RT). Weekly group meetings were established to ensure communication and close collaboration between the implementors, i.e., the key person, clinical nurse specialist, and first author (RT), to discuss the status of the program use and the monitoring of results, and to adjust the plan for the education and training of the nursing staff if necessary. Regular meetings between the first author and the nurse manager were planned to ensure active involvement and support. Themes for these meetings were the progress of the project and how the nurse manager could support it. The roles and tasks associated with the implementation were described for the nurse manager, the key person, and the clinical nurse specialist.

## Step 6: evaluation of the intervention program

### Monitoring and adjustment of the program

During the three-month intervention period, we continuously monitored and observed the healthcare professionals’ delivery of the ENI to identify additional needs for education and training. Initially, we focused on facilitating the nursing staff’s delivery of standard nutritional care, such as nutritional screening, calculations, and the entry of energy and protein requirements into the Food’n’Go system. Completion of these tasks were prerequisites for delivery of the ENI, and thus patients’ participation using Food’n’Go. Evaluation of the delivery of the ENI halfway through the intervention period showed a lack of patient participation. Based on our logic model of change, we assumed that we needed to intensify education targeting nursing staff’s attitudes and skills. We increased training on patient involvement, for example, communication with patients about their food intake using Food’n’Go. To facilitate the nursing staff’s awareness of the ENI, we provided them with results on patients’ food intake, as we expected that seeing the impact of their efforts would motivate them.

### Plan for evaluation of the feasibility and effectiveness of the ENI

A plan for evaluating the feasibility and effectiveness of the ENI was created. The feasibility was evaluated according to three components of process evaluation: “context,” “fidelity,” and “mechanism of impact” [45]. These components can be described as (1) contextual factors related to delivery and/or intervention outcomes (i.e., availability of Food’n’Go to patients and proportion of patients receiving dietary counseling by a dietitian); (2) whether the ENI was delivered as intended (intervention fidelity); and (3) mechanism of impact of the ENI (i.e., patients’ knowledge, skills, and use of Food’n’Go, and acceptability as perceived by patients and healthcare professionals). We evaluated the *effectiveness* of the ENI according to the patients’ food intake (energy and protein) after a three-month intervention period, using a pre- and post-test design. The evaluation will be reported elsewhere, except for acceptability of the intervention as perceived by the nursing staff, nurse manager, and dietitian, which is reported below.

## Part C: acceptability of the intervention as perceived by healthcare professionals

### Method

After the three-month intervention period, the healthcare professionals’ acceptability of the ENI, the education, and the support received were evaluated. This evaluation was based on two focus group interviews with nursing staff (n = 8) and two individual interviews with the nurse manager and dietitian affiliated with the unit during the intervention period. Written informed consent was obtained from all participants. We developed and used an interview guide addressing five components from the theoretical framework of acceptability by Sekhorn et al. [46]: a**ffective attitude** (how the participants feel about the intervention program); p**erceived effectiveness** (the extent to which the ENI is perceived to have achieved its intended purpose); s**elf-efficacy** (participants’ confidence in delivering the ENI); b**urden** (the amount of effort required to deliver the ENI); and i**ntervention coherence** (the extent to which the participants understand the ENI and how it works). These acceptability components were used when analyzing the qualitative data using deductive content analysis [47, 48].

### Results

#### Affective attitude

In general, the focus group interviews with nursing staff revealed a positive attitude toward the ENI based on their perception of the importance of nutrition for all patients, awareness of the consequences of malnutrition, and the importance of addressing these in nursing care. The nurse manager perceived a changed attitude among the nursing staff from a focus on barriers (e.g., patients’ lack of ability to use and benefit from eHealth), to a broader understanding of the involvement of patients in using Food’n’Go, and generally, an attitude toward nutrition as important. The nurse manager expressed that one possible cause for this changed attitude could be the increased focus on the essence of nursing —that is, patient outcomes (nutritional intake)—rather than on whether the nutritional screening procedure was performed in a timely manner. She felt that this motivated the nursing staff. A change in attitude and behavior among nursing staff was also experienced by the dietician, who believed that this change manifested itself in a stronger focus on nutrition, a higher degree of completion of required nutritional procedures (e.g., screening for malnutrition), and an increased awareness of providing patients with food and drinks with a high energy and protein content.

With regard to the entire intervention program, the nurse manager felt that her involvement in the development of the ENI had provided her with a broader perspective on opportunities for involving older patients in nutritional care when providing them with the necessary support. The nurse manager’s experience of being involved facilitated a sense of ownership of the project, which motivated her and made it easier for her to support it as leader.

#### Perceived effectiveness

The nursing staff described several examples of positive effects on patients’ nutritional intake when using the ENI. One example was some patients’ increased motivation to eat more when they, in cooperation with nursing staff, registered their own nutritional intake using Food’n’Go and received feedback showing their intake in relation to their needs. The effect of providing patients with information on nutrition was also discussed by the nursing staff. They observed that the patients’ choice of food was affected by the information and counseling they received, although they stated that many patients were still unaware of the importance of nutrition. The dietician also perceived Food’n’Go as useful for facilitating patient participation and thus beneficial, as it enhanced patients’ motivation and awareness of eating sufficiently. For example, the use of feedback on their dietary intake, visualized in a diagram, substantialized their goal for nutritional intake and motivated some patients to eat more to reach it. According to the dietician, the intervention program had also led to increased identification of patients at risk of malnutrition and, as a consequence, increased referrals to dietitians. This resulted in them being more present in the unit, which they felt had led to strengthened interdisciplinary collaboration.

#### Self-efficacy

Regarding the nursing staff’s perceived acceptability of the education and support received in the intervention period, they reported feeling confident in conducting the ENI. They felt that the knowledge and skills they had learned supported them. It was also emphasized, however, that they had limited experience with this new practice, and they were concerned about the prioritization of the ENI in competition with other tasks when time constraints were an issue. The combination of theory and practice was perceived as effective by the nursing staff. In particular, bedside teaching with skills training followed by individual feedback was perceived as beneficial for increasing their confidence in delivering the ENI. The nurse manager shared this opinion, and felt that the practical bedside teaching and skills training had had a positive impact on nursing staff’s motivation and learning.

#### Burden

The time required to support patients in the use of Food’n’Go was perceived as a burden by some nursing staff, as extra time was sometimes needed for different reasons. Regarding the perception of patient involvement using Food’n’Go as a burden due to time constraints, the nurse manager reflected on the importance of the management team emphasizing nutrition as a priority task. From the dietician’s perspective, no challenges with the use of Food’n’Go were experienced.

#### Intervention coherence

In general, the nursing staff and the dietitian seemed to understand the ENI and how supporting the patients to participate in nutritional care using Food’n’Go might motivate them to eat sufficiently. However, the focus group interviews revealed that not all nursing staff were aware of how to provide individualized support to patients, as described in the ENI. Some had missed that the involvement of patients in the use of Food’n’Go should be differentiated and target the patients’ individual competence, or were unaware of how to do it. In some cases, unnecessary effort was made to get patients without sufficient competence and ability to hold and operate the tablet themselves. Since this was often unsuccessful, patient involvement was perceived as a time constraint and a burden, as described above.

## Discussion

We have presented how we developed an intervention program aimed at supporting older patients to participate in their own nutritional care using the eHealth solution Food’n’Go, guided by IM. The intervention program consists of (1) the ENI, and (2) a plan for education and training targeting nursing staff. The process, data, resulting intervention, and documented acceptability are unique in that they directly address the specific challenge: How can patient participation and the newly introduced technology “Food’n’Go” be adopted and embedded in everyday practice?

Acutely ill older patients are often frail and depend on support to manage their own nutrition, including use of eHealth which may explain the common belief that older patients are incapable of using and benefiting from eHealth [49, 50]. The initial steps in IM (steps 1 and 2) made it clear that a broader understanding of the use of eHealth among nursing staff was necessary in the context of hospitalized older patients. We identified varying levels of ICT competence, which indicated a need for differentiated support; consequently, the four levels of user competence and need for support we identified were addressed in the ENI. This allowed us to differentiate the involvement of the patients in nutritional care using Food’n’Go.

In step 1, the needs assessments showed that in addition to patients, healthcare professionals and relatives were also important target groups to address in the intervention. Nutrition is described as a core area of nursing care [51], which contrasts with the insufficient delivery of nutritional care that we identified, and which is also reported in several other studies [19, 32]. In alignment with Noort et al., who developed a nutritional intervention for an anesthesia outpatient clinic [19], we developed a program for education and training for nursing staff to ensure intervention delivery. The logic models (steps 1 and 2) emphasized that in addition to the nutrition perspective, it was important to enhance nursing staff’s knowledge, skills, and attitudes regarding patient involvement. Patient involvement is often limited to “providing information” [52]. Therefore, to ensure successful implementation, it is important to recognize that patient involvement requires specific knowledge and skills to be acquired by nursing staff.

In step 3, IM guided us to develop an intervention with the systematic use of relevant theory and evidence, considering the specific context of older hospital patients. IM has been described as a framework that can be used to bridge the gap between theory and practice [53]. Developing the intervention map in a sociotechnical context illustrated how a combination of theories from different areas, such as SCT, eHealth literacy, and technology acceptance, together with empirical data, can inform a systematic design process. Adoption and successful use of eHealth solutions require that end users are capable and motivated to use these technologies [54], so the integration of theories about technology acceptance, readiness, and eHealth literacy is important in the development of an intervention such as the ENI. A systematic reviews concerning eHealth literacy interventions for older people found that most existing interventions lacked a theoretical framework of eHealth literacy [54]. In the development of the ENI, we used a conceptual theory-based framework of eHealth literacy, which captures dimensions related to users’ knowledge and skills, their motivation and trust in technology, and context, combined with the theory of technology acceptance [34, 55]. We expected that addressing these theory-based eHealth literacy dimensions in the ENI would facilitate the adaption and successful use of Food’n’Go and thereby have a positive impact on the outcome.

In steps 3 and 4, we involved end users and relevant stakeholders by inviting them to come up with ideas and provide feedback on planned concepts and information materials. Collaborative involvement of end users is important for capturing their perspectives on problems and ensuring the acceptance and successful implementation of a new system [43, 56]. Researchers have argued that the involvement of end users should take place in the very early stages of the design and development of interventions, and that end users should not be limited to the role of informants [43, 57]. We involved older patients and their relatives to include their ideas and perspectives on the presented concepts.

As part of steps 5 and 6, we decided to continuously evaluate parameters that were prerequisites for delivering the ENI as well as its outcomes (nutritional intake). These evaluations informed adjustments to the teaching to support nursing staff in their needs, thus facilitating the implementation and maintenance of the ENI. Boonestra et al. argued that providing users with the necessary support and prompt responses to their requests during the implementation of a system would enhance their motivation, which is important for their acceptance of a new eHealth solution [58]. These adjustments may have contributed to the nursing staff’s acceptance of the intervention.

The acceptability of the intervention as perceived by the healthcare professionals was part of the evaluation, which we planned in step 6. By understanding and addressing healthcare professionals’ beliefs and attitudes, we were able to create an intervention that had high overall acceptability and was anticipated to provide a higher level of patient participation in nutritional care assisted by an eHealth solution. The evaluation revealed a changed attitude among nursing staff toward a more positive attitude regarding the involvement of the patients in nutritional care using Food’n’Go. The dietician experienced positive impacts of this changed attitude in the nutritional care provided by the nursing staff, including a higher degree of completion of nutritional screening and an increased focus on how to meet patients’ nutritional needs. This positive change was explained by the nurse manager as a perception among the nursing staff that the education was meaningful due to the increased focus on patient outcomes (i.e., patients’ nutritional intake) and the use of bedside training. Consistent with SCT, skills training was a way to increase self-efficacy [38, 39]. Self-efficacy is an important determinant to address, as according to Bandura, it affects individuals’ behavior both directly and through its influence on other determinants [38].

The evaluation of acceptability also indicated some issues that must be taken into consideration in future implementation of the ENI. The use of Food’n’Go was sometimes perceived as a burden due to deficient knowledge and skills regarding how to differentiate the involvement of patients. The use of Food’n’Go became the goal instead of a means of reaching the goal of participation in their own nutrition. This indicated that we had not succeeded in providing all nursing staff with sufficient knowledge and skills to use Food’n’Go in a differentiated way. It emphasizes the importance of monitoring intervention fidelity to adjust the nursing staff-directed education and training. Furthermore, an implementation period longer than three months may be required for this training.

With regard to the overall design and development process of the intervention program, the use of IM made several advantageous contributions to this study. Guided by IM, we developed the intervention program in a dynamic iterative process, which is recommended when developing a complex intervention such as the ENI [59]. The widely used UK Medical Research Council’s (MRC) guidance for developing complex interventions has several similarities with IM, including the emphasis on developing theory- and evidence-based interventions, taking the dynamic relationship between the intervention and its context into account, and the importance of involving stakeholders [60]. The IM framework is inspired by the discipline of health education, which is important for our study as it involved the development of an educative intervention. Use of IM is a time-consuming process, as has been reported in other studies [19]. However, we argue that IM facilitates a stepwise systematic approach that enhances the possibility of designing and developing a complex intervention, taking the complexity of the local context and its end users into account.

This study may have some limitations. When using a sociotechnical approach, the technology is seen as an actor in itself with a specific influence on its use, and is therefore also an environmental factor, which we included in the needs assessment. Nevertheless, ongoing adjustments to the Food’n’Go system in response to needs were not part of the development of this intervention program. A prior study showed that older people living at home found the Food’n’Go technology acceptable and generally easy to use [15]. The ENI was designed and developed tailored to be used in one unit in a university hospital. Consequently, the ENI may not be transferrable to another context without adjustments tailored to the new context [59, 61].

In conclusion, guided by the IM framework and a sociotechnical approach, we have developed a theory- and evidence-based ENI that is expected to facilitate an environment that supports older patients to actively participate in their own nutritional care, assisted by eHealth. Overall, the acceptability as perceived by nursing staff, the nurse manager, and the dietician were high.

## Electronic supplementary material

Below is the link to the electronic supplementary material.

Supplementary Material 1

Supplementary Material 2

## Data Availability

Data containing personal information cannot be disclosed due to the rules of the General Data Protection Regulation, but author RT can be contacted about any possibilities of seeing aggregated or anonymized parts of the dataset.

## Abbreviations

HER: Electronic health record
ENI: Educative nutritional intervention
ESPEN: European Society for Clinical Nutrition and Metabolism
ICT: Information and communication technology
IM: Intervention mapping
LPN: Licensed practical nurses
NRS: Nutritional risk screening
PO: Performance objectives
READHY: Readiness and enablement index for health technology
RN: Registered nurse
SCT: Social cognitive theory
TAM3: Technology acceptance model 3

## References

[1] Leij-Halfwerk S, Verwijs MH, van Houdt S, Borkent JW, Guaitoli PR, Pelgrim T (2019). Prevalence of protein-energy malnutrition risk in European older adults in community, residential and hospital settings, according to 22 malnutrition screening tools validated for use in adults ≥ 65 years: A systematic review and meta-analysis. Maturitas.

[2] Volkert D, Beck AM, Cederholm T, Cereda E, Cruz-Jentoft A, Goisser S (2019). Management of Malnutrition in Older Patients—Current Approaches, Evidence and Open Questions. J Clin Med.

[3] Sharma Y, Miller M, Kaambwa B, Shahi R, Hakendorf P, Horwood C (2017). Malnutrition and its association with readmission and death within 7 days and 8-180 days postdischarge in older patients: a prospective observational study. BMJ Open.

[4] Felder S, Lechtenboehmer C, Bally M, Fehr R, Deiss M, Faessler L (2015). Association of nutritional risk and adverse medical outcomes across different medical inpatient populations. Nutrition.

[5] Agarwal E, Ferguson M, Banks M, Batterham M, Bauer J, Capra S (2013). Malnutrition and poor food intake are associated with prolonged hospital stay, frequent readmissions, and greater in-hospital mortality: results from the Nutrition Care Day Survey 2010. Clin Nutr.

[6] Covinsky KE, Martin GE, Beyth RJ, Justice AC, Sehgal AR, Landefeld CS (1999). The relationship between clinical assessments of nutritional status and adverse outcomes in older hospitalized medical patients. J Am Geriatr Soc.

[7] Malafarina V, Reginster JY, Cabrerizo S, Bruyère O, Kanis JA, Alfredo Martinez J (2018). Nutritional status and nutritional treatment are related to outcomes and mortality in older adults with hip fracture. Nutrients.

[8] Sorensen J, Kondrup J, Prokopowicz J, Schiesser M, Krähenbühl L, Meier R (2008). EuroOOPS: an international, multicentre study to implement nutritional risk screening and evaluate clinical outcome. Clin Nutr.

[9] Munk T, Tolstrup U, Beck AM, Holst M, Rasmussen HH, Hovhannisyan K (2016). Individualised dietary counselling for nutritionally at-risk older patients following discharge from acute hospital to home: A systematic review and meta-analysis. J Hum Nutr Diet.

[10] Baldwin C, Weekes CE. Dietary advice for illness-related malnutrition in adults. Cochrane Database Syst Rev. 2008;23(1):CD002008. 10.1002/14651858.CD002008.pub3 18254000

[11] Collins J, Porter J (2015). The effect of interventions to prevent and treat malnutrition in patients admitted for rehabilitation: a systematic review with meta-analysis. J Hum Nutr Diet.

[12] Beck AM, Dent E, Baldwin C (2016). Nutritional intervention as part of functional rehabilitation in older people with reduced functional ability: a systematic review and meta-analysis of randomised controlled studies. J Hum Nutr Diet.

[13] Volkert D, Beck AM, Cederholm T, Cruz-Jentoft A, Goisser S, Hooper L (2019). ESPEN guideline on clinical nutrition and hydration in geriatrics. Clin Nutr.

[14] Prey JE, Woollen J, Wilcox L, Sackeim AD, Hripcsak G, Bakken S (2014). Patient engagement in the inpatient setting: A systematic review. J Am Med Inform Assoc.

[15] Lindhardt T, Nielsen MH (2017). Older patients’ use of technology for a post-discharge nutritional intervention – A mixed-methods feasibility study. Int J Med Inform.

[16] Bartholomew LK, Markham CM, Ruiter RAC, Fenandez ME, Kok G, Parcel GS. Planning Health Promotion Programs: An Intervention Mapping Approach, 4 ed. San Francisco: Jossey-Bass Inc., 2016:1–704.

[17] Dalager T, Højmark A, Jensen PT, Søgaard K, Andersen LN (2019). Using an intervention mapping approach to develop prevention and rehabilitation strategies for musculoskeletal pain among surgeons. BMC Public Health.

[18] Hesselink G, Zegers M, Vernooij-Dassen M, Barach P, Kalkman C, Flink M (2014). Improving patient discharge and reducing hospital readmissions by using Intervention Mapping. BMC Health Serv Res.

[19] Van Noort HHJ, Heinen M, Van Asseldonk M, Ettema RGA, Vermeulen H, Huisman-De Waal G (2020). Using intervention mapping to develop an outpatient nursing nutritional intervention to improve nutritional status in undernourished patients planned for surgery. BMC Health Serv Res.

[20] Van Dongen EJ, Leerlooijer JN, Steijns JM, Tieland M, De Groot LC, Haveman-Nies A (2017). Translation of a tailored nutrition and resistance exercise intervention for elderly people to a real-life setting: adaptation process and pilot study. BMC Geriatr.

[21] van Dulmen S, Driesenaar JA, van Weert JCM, van Osch M, Noordman J, PatientVOICE (2017). Development of a preparatory, pre-chemotherapy online communication tool for older patients with cancer. JMIR Res Protoc.

[22] Chang SJ, Yang E, Lee KE, Ryu H. Internet health information education for older adults: A pilot study. Geriatr Nurs. 2021;(2):533–539. 10.1016/j.gerinurse.2020.10.002 33092906

[23] Kitson A, Conroy T, Wengstrom Y, Profetto-McGrath J, Robertson-Malt S. Defining the fundamentals of care. Int J Nurs Pract. 2010;(4):423–34. 10.1111/j.1440-172X.2010.01861.x 20649678

[24] Sauer AC, Alish CJ, Strausbaugh K, West K, Quatrara B. Nurses needed: Identifying malnutrition in hospitalized older adults. NursingPlus Open 2. 2016;21–25.

[25] Kondrup J, Ramussen HH, Hamberg O, Stanga Z, Camilo M, Richardson R (2003). Nutritional risk screening (NRS 2002): A new method based on an analysis of controlled clinical trials. Clin Nutr.

[26] Kok G, Peters LWH, Ruiter RAC (2017). Planning theory- and evidence-based behavior change interventions: A conceptual review of the intervention mapping protocol. Psicol. Refl. Crit.

[27] Agarwal E, Marshall S, Miller M, Isenring E (2016). Optimising nutrition in residential aged care: A narrative review. Maturitas.

[28] Volkert D (2013). Malnutrition in older adults-urgent need for action: A plea for improving the nutritional situation of older adults. Gerontology.

[29] Avgerinou C, Bhanu C, Walters K, Croker H, Liljas A, Rea J (2019). Exploring the views and dietary practices of older people at risk of malnutrition and their carers: A qualitative study. Nutrients.

[30] Heersink JT, Brown CJ, Dimaria-Ghalili RA, Locher JL (2010). Undernutrition in hospitalized older adults: patterns and correlates, outcomes, and opportunities for intervention with a focus on processes of care. J Nutr Elder.

[31] Terp R, Kayser L, Lindhardt T (2021). “It is not rocket science.” – Older peoples’ understanding of nutrition – A qualitative study. Appetite.

[32] Beck AM, Knudsen AW, Østergaard TB, Rasmussen HH, Munk T (2021). Poor performance in nutrition risk screening may have serious consequences for hospitalized patients. Clin Nutr ESPEN.

[33] Venkatesh V, Bala H (2008). Technology acceptance model 3 and a research agenda on interventions. Decis Sci.

[34] Kayser L, Rossen S, Karnoe A, Elsworth G, Vibe-Petersen J, Christensen JF (2019). Development of the multidimensional Readiness and Enablement Index for health Technology (READHY) tool to measure individuals’ health technology readiness: Initial testing in a cancer rehabilitation setting. J Med Internet Res.

[35] Terp R, Kayser L, Lindhardt T (2021). Older Patients’ Competence, Preferences, and Attitudes Toward Digital Technology Use: Explorative Study. JMIR Hum Factors.

[36] Van Houwelingen CTM, Ettema RGA, Antonietti MGEF, Kort HSM (2018). Understanding older people’s readiness for receiving telehealth: Mixed-method study. J Med Internet Res.

[37] Volkert D, Kiesswetter E, Cederholm T, Donini LM, Eglseer D, Norman K (2019). Development of a Model on Determinants of Malnutrition in Aged Persons: A MaNuEL Project. Gerontol Geriatr Med.

[38] Bandura A. On the functional properties of perceived self-efficacy revisited. Journal of Management. 2012;38(1):9–44.

[39] Kelder S, Hoelscher D, Perry CL (2015). How individuals, environments and health behaviors interact: Social cognitive theory. Health behavior: Theory, research, and practice.

[40] Michie S, Johnston M, Francis J, Hardeman W, Eccles M (2008). From Theory to Intervention: Mapping Theoretically Derived Behavioural Determinants to Behaviour Change Techniques. Appl Psychol.

[41] Kok G, Gottlieb NH, Peters GJY, Mullen PD, Parcel GS, Ruiter RAC (2016). A taxonomy of behaviour change methods: an Intervention Mapping approach. Health Psychol Rev.

[42] Perloff RM. The dynamics of persuasion: Communication and attitudes in the 21st century. The Dynamics of Persuasion: Communication and Attitudes in the Twenty-First Century. Rooutledge New York. 2010;p.40–183.

[43] Kensing F, Blomberg J (1998). Participatory Design: Issues and Concerns. Comput Support Coop Work.

[44] Blair W, Smith B (2012). Nursing documentation: Frameworks and barriers. Contemp Nurse.

[45] Moore GF, Audrey S, Barker M, Bond L, Bonell C, Hardeman W (2015). Process evaluation of complex interventions: Medical Research Council guidance. BMJ.

[46] Sekhon M, Cartwright M, Francis JJ (2017). Acceptability of healthcare interventions: An overview of reviews and development of a theoretical framework. BMC Health Serv Res.

[47] Graneheim UH, Lundman B (2004). Qualitative content analysis in nursing research: concepts, procedures and measures to achieve trustworthiness. Nurse Educ Today.

[48] Elo S, Kyngäs H (2008). The qualitative content analysis process. J Adv Nurs.

[49] Ramirez-Zohfeld V, Seltzer A, Xiong L, Morse L, Lindquist LA (2020). Use of Electronic Health Records by Older Adults, 85 Years and Older, and Their Caregivers. J Am Geriatr Soc [Internet].

[50] Arthanat S, Vroman KG, Lysack C, Grizzetti J (2019). Multi-stakeholder perspectives on information communication technology training for older adults: implications for teaching and learning. Disabil Rehabil Assist Technol.

[51] Feo R, Kitson A, Conroy T (2018). How fundamental aspects of nursing care are defined in the literature: A scoping review. J Clin Nurs.

[52] Menichetti J, Graffigna G, Steinsbekk A (2018). What are the contents of patient engagement interventions for older adults? A systematic review of randomized controlled trials. Patient Educ Couns.

[53] Garba RM, Gadanya MA (2017). The role of intervention mapping in designing disease prevention interventions: A systematic review of the literature. PLoS ONE.

[54] Kushniruk AW, Bates DW, Bainbridge M, Househ MS, Borycki EM (2013). National efforts to improve health information system safety in Canada, the United States of America and England. Int J Med Inform.

[55] Kayser L, Karnoe A, Furstrand D, Batterham R, Christensen KB, Elsworth G (2018). A Multidimensional Tool Based on the eHealth Literacy Framework: Development and Initial Validity Testing of the eHealth Literacy Questionnaire (eHLQ). J Med Internet Res.

[56] Kensing F, Sigurdardottir H, Stoop A (2007). MUST-A participatory method for designing sustainable health IT. Stud Health Technol Inform.

[57] Clemensen J, Rothmann MJ, Smith AC, Caffery LJ, Danbjorg DB (2017). Participatory design methods in telemedicine research. J Telemed Telecare.

[58] Boonstra A, Versluis A, Vos JFJ (2014). Implementing electronic health records in hospitals: A systematic literature review. BMC Health Serv Res.

[59] Greenhalgh T, Papoutsi C (2018). Studying complexity in health services research: Desperately seeking an overdue paradigm shift. BMC Med.

[60] Craig P, Dieppe P, Macintyre S, Mitchie S, Nazareth I, Petticrew M (2008). Developing and evaluating complex interventions: The new Medical Research Council guidance. BMJ.

[61] Moore G, Campbell M, Copeland L, Craig P, Movsisyan A, Hoddinott P (2021). Adapting interventions to new contexts-the ADAPT guidance. BMJ.

