# Supplementary material for: Theory-driven development of an educative nutritional intervention (ENI) supporting older hospital patients to eat sufficiently, assisted by an eHealth solution: an intervention mapping approach
Source: BMC Health Serv Res. 2022 Nov 28;22:1435. doi: 10.1186/s12913-022-08679-8 (PMC9706976; doi:10.1186/s12913-022-08679-8)
Supplement: Supplementary file 1 — Supplementary Material 1 [file 12913_2022_8679_MOESM1_ESM.pdf]

Additional file 1. Complete matrices of change objectives for patients, relatives, and healthcare professionals (step 2)

**Performance objectives (PO) for patients**	**Change objectives**					
	**Knowledge**	**Skills**	**Self-efficacy**	**Outcome expectation**	**Social support**	**Attitude and awareness**
**Profile 1 and 2***<br>PO.1.1<br>**Use of Food'n'Go**<br>Operate the tablet and use Food'n'Go<br>• Order food and drinks<br>• Register their food intake**<br>• Monitor and adjust their food intake if necessary	Express the routines with regard to using Food'n'Go to order food and register their intake – when and how	Demonstrate skills to operate Food'n'Go to:<br>• Order food<br>• Register intake<br>• Adjust food intake based on feedback from Food'n'Go to ensure an adequate dietary intake	Express confidence in ordering food and registering their food intake in Food'n'Go	Expect Food'n'Go to provide options to choose tasty foods that will meet their preferences and dietary requirements<br><br>Expect the healthcare professionals and relatives approve that they use Food'n'Go	Perceive support from their relatives in using Food'n'Go<br><br>Perceive that the nursing staff provide them with necessary support with regard to using Food'n'Go<br><br>Ask the nursing staff for help when needed with regard to Food'n'Go	Perceive Food'n'Go to be useful and easy to use
**Profile 3***<br>PO.1.2<br>**Use of Food'n'Go**<br>Participate in the use of Food'n'Go in cooperation with the nursing staff who hold and operate the tablet			Express confidence in ordering food and drinks and registration of their intake together with the nursing staff who hold the tablet	Expect Food'n'Go to provide options to choose tasty foods that will meet their preferences and dietary requirements<br><br>Expect the healthcare professionals and relatives to approve that they participate in using Food'n'Go	Perceive support from their relatives in using Food'n'Go<br><br>Perceive that the nursing staff provides them with necessary support with regards to using Food'n'Go	Perceive Food'n'Go to be useful and easy to use
**Profiles 1, 2, and 3***<br>PO.2.1<br>Order and eat food that meets their nutritional needs	Express their nutritional needs<br><br>Express how a sufficient food intake positively affects their health, including physical functioning<br><br>Express they need food and drink with high content of energy and protein	Demonstrate they use the feedback on their daily food intake provided in Food'n'Go and adjust their food intake during the day, if necessary		Expect that eating sufficiently will positively affect their recovery and physical function<br><br>Expect that they will be served well-prepared and tasty food<br><br>Expect the healthcare professionals to approve they eat sufficiently during their hospital	Perceive support from their relatives and are engaged in the effort of eating sufficiently.<br><br>Perceive that the healthcare professionals will provide them with necessary support with regards to selecting food that meets their needs	Aware of the importance of eating despite no or decreased appetite.

PO. 2.2<br>Order and eat food and drinks with high content of energy and protein despite experiencing decreased appetite<br><br>PO. 2.3<br>Order and Eat in-between meals 2-3 times a day	Indentify food and drink items with high content of protein and energy<br><br>Describe when and how to order and be served in-between meals	**Only profiles 1 and 2:**<br>Demonstrate how to order food and drinks with high content of energy and protein on the tablet (Food'n'Go)<br><br>Demonstrate how to order in-between meals		stay<br><br>Expect that consuming food and drinks with high content of energy and protein will be an easier way to reach their nutritional needs when they have no appetite<br><br>Expect that eating between meals is an important way to cover their daily needs of energy and protein and thus important for their recovery and physical function<br><br>Expect they will be served tasty in-between meals that cover their nutritional needs.	Perceive that their relatives are engaged and support them in the effort of eating sufficiently<br><br>Perceive that the nursing staff will provide them with the necessary support with regards to serving the food and drinks that suit their needs<br><br>Perceive that their relatives are engaged and support them in the effort of eating sufficient<br><br>Perceive the nursing staff will provide them with necessary support with regards to serving the food and drink they ask for	Aware of ordering in-between meals twice a day from the mobile trolley
**Performance objectives for relatives**	**Change objectives**					
	**Knowledge**	**Skills**	**Self-efficacy**	**Outcome expectation**	**Social support**	**Attitude and awareness**
Participate and support the patients in the nutritional intervention by:<br><br>• Talking with the patient about their food intake<br>• Encourage and assist the patient in the use of Food'n'Go<br>• Encourage the patient to order and eat in-between meals<br>• Keep patients company during meals	Describe nutritional needs when ill and older<br><br>Understand the department's routines and offering with regards to Food'n'Go:<br><br>• How and when to order food using Food'n'Go<br>• How to register intake of food and drinks<br>• How to receive feedback on daily food intake	Demonstrate how to use Food'n'Go<br><br>*(Relatives' use of the patients' tablet requires an informed consent form from the patient.)*		Expect that their involvement in the nutritional intervention will have a positive effect on the patient's food intake		Express an attitude reflecting:<br><br>Food'n'Go is easy to use<br><br>Older people are capable of using and benefiting from Food'n'Go when they receive the necessary support

	Express the department's offering for in-between meals<br>Express how they can motivate the patient to eat better					
**Performance objectives for healthcare professionals**	**Change objectives**					
	**Knowledge**	**Skills**	**Self-efficacy**	**Outcome expectation**		**Attitude and awareness**
Nursing staff:<br><br>• Assess whether patients can apply and benefit from Food'n'Go<br>• Inform and introduce eligible patients to Food'n'Go<br>• Inform and counsel patients about nutrition<br>• Involve patients in the use of Food'n'Go according to patient profiles during hospitalization<br>• Give personal feedback to patients about their nutritional intake during hospitalization<br>• Involve the relatives in the nutritional interventions when relevant<br>• Inform the interdisciplinary team about patients at risk of malnutrition	Describe nutritional needs when ill and older<br><br>Understand how to<br>• Assess the patients' needs for support to use Food'n'Go<br>• Inform and introduce patients to Food'n'Go<br>• Inform and counsel on nutrition<br>• Involve patients in the use of Food'n'Go<br>• Give personal feedback on nutritional intake using Food'n'Go<br>• Involve relatives in the nutritional interventions<br>• Coordinate interdisciplinary nutrition care	Demonstrate skills to use Food'n´Go and the website<br><br>Demonstrate skills to involve the patients and their relatives in the nutritional intervention:<br><br>• Use of Food'n'Go<br>• Information and counseling<br><br>Demonstrate skills to coordinate interdisciplinary nutrition care<br><br>Demonstrate skills to document the patients' needs for support in use of Food'n'Go in the EPJ	Express confidence in involving the patients in the nutritional intervention using Food'n'Go	Expect that systematic involvement of patients and relatives in the nutritional intervention by using Food'n'Go will motivate the patients to eat better		Express an attitude reflecting:<br><br>Older people are capable of using and benefiting from Food'n'Go when they receive the necessary support.<br><br>Prioritization of nutrition is extremely important in older patients.<br><br>Food'n'Go is easy to use and a useful tool for involving patients in their nutrition.<br><br>Involving patients and relatives in the nutritional intervention is feasible in daily practice.
Dietitians:<br><br>Use Food'n'Go in dietary counseling	Understand how to involve the patients by using Food'n'Go when dietary counseling	Demonstrate use of Food'n´Go and can operate in the system		Expect that use of Food'n'Go will enhance the patients' involvement and motivation to adhere to		

				their dietary advices		
Physicians:<br>Addresses the importance of sufficient food intake in conversations with patients at risk of malnutrition during ward rounds	Know the workflow, criteria and the physicians' role regarding informing patients about nutrition			Expect older patients to adhere to advice on nutrition provided by the physicians		Pay attention to nutritional issues at ward rounds among patients at risk of malnutrition i.e. address the importance of sufficient nutrition

* Profile 1: Patient is able to hold and operate the tablet with Food'n'Go without support.
Profile 2: Patient is able to hold and operate the tablet with Food'n'Go with verbal and/or technical support.
Profile 3: Patient is able to participate in completing the tasks when the tablet with Food'n'Go is held and operated by another.
