# Supplementary material for: Theory-driven development of an educative nutritional intervention (ENI) supporting older hospital patients to eat sufficiently, assisted by an eHealth solution: an intervention mapping approach
Source: BMC Health Serv Res. 2022 Nov 28;22:1435. doi: 10.1186/s12913-022-08679-8 (PMC9706976; doi:10.1186/s12913-022-08679-8)
Supplement: Supplementary file 2 — Supplementary Material 2 [file 12913_2022_8679_MOESM2_ESM.pdf]

Additional file 2. Intervention program with theory-based change methods and practical applications (step 3): Comprehensive version of Table 2

Change objectives	Theory-based change methods	Practical application and delivery
**PATIENTS**		
**Knowledge**<br>• Food intake and health<br>• Nutritional needs when older and ill<br>• Food and drink items containing high levels of energy and protein<br>• Nutritional care while hospitalized, including Food'n'Go<br>• Routines with regards to meals and food—when and how to order	Provide information [a,]	Posters positioned at the entrances to the unit and at the nurses' station<br><br>Pamphlet on nutrition handed out to patients and relatives<br><br>Individual communication with the nursing staff and dietitians<br><br>Food'n'Go user guide placed on the patients' whiteboards<br><br>Information about the content of energy and protein in food and drinks on Food'n'Go system
**Skills**<br>*(Only patient profile 1 and 2)*<br><br>Use of Food'n'Go	Guided practice [b, c]<br><br>Goal and self-monitoring [a, b, c]	The nursing staff instructs patients in how to use Food'n'Go on the day of admission, if possible, or next day before lunch. Procedures and teaching principles for instructions are outlined in the guideline "Involvement of patients and relatives in the nutritional intervention."<br><br>Patients at risk of malnutrition (NRS score ≥ 3) are introduced and taught to monitor their dietary intake and use the feedback provided in the Food'n'Go system.
**Self-efficacy**<br>Feel capable to use Food'n'Go	Guided practice [b, c]<br><br>Encourage and provide feedback on their performance [c]	Patients are instructed and supported in the use of Food'n'Go in accordance with their competencies and need for support (according to the four defined patient profiles) and supported toward a mastery experience.<br><br>The nursing staff and dietitians use Food'n'Go in guidance and counseling.
**Outcome expectation**<br>The beneficial effect of eating adequately and use of Food'n'Go<br><br>Expect approval from relatives and healthcare professionals when eating adequately and using Food'n'Go	Provide information on consequences and outcome [a]<br><br>Persuasive communication [a, d]<br><br>Social processes of encouragement [a]	Verbal and written information communicates key messages about potential beneficial outcomes of eating sufficiently and using Food'n'Go.<br><br>The physicians inform and talk about nutrition during ward rounds among patients at risk of malnutrition.<br><br>The healthcare professionals recognize the patients' efforts to use Food'n'Go and eat sufficiently.
**Social support**<br>Feel that relatives support them in the effort to eat sufficiently using Food'n'Go	Social processes of encouragement and support [a, c]	Support is sought from relatives by providing the them with knowledge etc. (see below under "Relatives").

Feel they are provided the necessary support from the healthcare professionals		
**Attitude and awareness**<br>• Perceive Food'n'Go to be easy to use and useful<br>• Order and consume food and drinks despite lack of appetite	Persuasive communication [a, d]<br><br>Cues [a]<br><br>Self-monitoring [a]	Providing patients with the knowledge and skills described above is expected to influence their attitude and awareness.<br><br>Patients at risk of malnutrition are offered individual dietary counseling by a dietitian.<br><br>Magnets on the patients' whiteboard visualize the expected behavior regarding the use of Food'n'Go.<br><br>Food'n'Go enables self-monitoring with regards to patient's nutritional intake in comparison to their nutritional needs.
**RELATIVES**		
**Knowledge**<br>• Nutritional needs when older and ill<br>• Possibilities and routines with regards to meals and food ordering—when and how<br>• The Food'n'Go technology<br>• How relatives can support and motivate the patient to eat sufficiently	Provide information [a]	Written information (posters and pamphlets with key messages about nutrition and a user guide for Food'n'Go) and verbal information are provided by the nursing staff.<br><br>Note: the relatives' use of Food'n'Go
**Outcome expectation**<br>Adequate dietary intake will positively influence physical functioning in older people.	Provide information on consequences and outcome [a]	Providing the information as described above under "knowledge"
**Attitude**<br>Perceive Food'n'Go to be an easy-to-use and useful tool<br><br>Older people are capable of using and benefiting from Food'n'Go when they receive the necessary support.	Persuasive communication [a, d]	Providing the relatives with the above information is expected to influence their attitude.<br><br>During personal communication, the nursing staff emphasize the importance of an adequate intake in older people and the benefits of using Food'n'Go.
**Nursing staff**	**Activities provided during intervention period to facilitate implementation**	
**Knowledge**<br>• Nutritional knowledge<br>• Assessment of patients' needs of support to use Food'n'Go<br>• Involvement of patients and relatives in nutrition and use of Food'n'Go<br>• Coordination of interdisciplinary nutrition care	Provide information [a]	Teaching sessions once a week with different topics related to the ENI<br><br>Individual teaching sessions<br><br>Weekly electronic newsletter<br><br>Guidelines targeted at the nursing staff describing procedure for conducting the ENI<br><br>Posters with key messages related to the ENI formulated as the "10 nutrition commandments"

**Skills and self-efficacy**<br>• Use of Food'n'Go and the back-end website<br>• Assessment of patients' needs for support<br>• Involvement of patients and relatives<br>• Nursing documentation	Guided practice [b, c]<br><br>Modeling [a, c, e]<br><br>Encourage and provide feedback on their performance [c]	Individual bedside teaching is provided with skills training followed by feedback. Patients are supported toward a mastery experience.<br><br>The nutritional key person demonstrates use of the ENI in daily nursing care.<br><br>Results from the ongoing monitoring are relayed to the staff.
**Outcome expectation**<br>Involvement of patients and relatives in the nutritional intervention by using Food'n'Go will motivate the patients to eat better<br><br>Expect approval from the management when conducting the ENI	Provide information regarding outcome [a]<br><br>Encourage and provide feedback on their performance [c]	Weekly teaching sessions<br><br>Continuously provide the nursing staff with results from monitoring about the patients' use of Food'n'Go and their food intake<br><br>The nurse manager:<br>• Participates in meetings and teaching sessions related to the intervention to signify the importance.<br>• Requests action in relation to delivery of the ENI.
**Attitude and awareness**<br>Older patients are able to use and benefit from Food'n'Go.<br><br>It is a priority to involve the patients in the nutritional intervention using Food'n'Go.<br><br>Food'n'Go is easy to use and a useful tool for involving patients in their own nutrition.<br><br>Involving patients and relatives in the nutritional intervention using Food'n'Go is feasible in daily practice.	Persuasive communication [a, d]<br><br>Encourage and provide feedback on their performance [c]<br><br>Facilitation—reduce barriers to action [f]	High involvement is facilitated in teaching by providing the nursing staff with above information about nutrition and patient involvement and encourage discussion and reflection about the use of ENI during the weekly teaching sessions.<br><br>Continuous training of skills related to the ENI is expected to change their attitude.<br><br>Food'n'Go is available (charged and logged in) to all patients. Weekly checks of availability of Food'n'Go take place.
**Dietitians**	**Activities provided during intervention period to facilitate implementation**	
**Knowledge, skills, and self-efficacy**<br>Use of Food'n'Go and the back-end website	Provide information and instruction [a]<br><br>Persuasive communication [a, e]	Regular meetings take place with the dietitians and first author.<br><br>The dietitians participate in the weekly teaching sessions with the nursing staff.
**Outcome expectation**<br>Involvement of the patients using Food'n'Go will motivate the patients to adhere to the dietary counseling	Provide information regarding outcome [a]<br><br>Encourage and provide feedback on their performance [c]	
**Physicians**	**Activities provided during intervention period to facilitate implementation**	

**Knowledge**<br>Workflow related to management of patients at risk of malnutrition<br><br>**Outcome expectation**<br>Older patients tend to comply with advice from physicians<br><br>**Awareness**<br>Inform and encourage patients at risk of malnutrition	Provide information [a]	Information meetings are held before the start of the intervention.<br><br>At the morning meetings, the interdisciplinary staff are continuously informed and encouraged to perform the required tasks related to the ENI by the nurse manager and the first author.

[a]Mitchie et al., 2008 [40]; [b]Kok et al., 2016 [41]; [c]Kelder et al., 2016 [39]; [d]Perloff, 2017 [42]; [e] Bandura, 2012 [38]; [f] Bartholomew et al., 2016 [16]
